# Editorial: Cancer biology, immunotherapy and aging

**DOI:** 10.3389/fragi.2026.1776719

**Published:** 2026-01-20

**Authors:** Stephane Koda, Jensen G. Weedor, Parfait Botolo Sakava

**Affiliations:** 1 Department of Pathogen Biology and Immunology, Jiangsu Key Laboratory of Immunity and Metabolism, Jiangsu International Laboratory of Immunity and Metabolism, Xuzhou Medical University, Xuzhou, Jiangsu, China; 2 Department of Physiology, Xuzhou Medical University, Xuzhou, Jiangsu, China

**Keywords:** aging, cancer biology, data-driven methods, immunotherapy, tumor microenvironment

## 1 Introduction

Cancer biology, immunotherapy, and aging converge in ways that challenge traditional clinical paradigms (Ontiveros et al., 2023; Kao et al., 2025). As the immune system undergoes age-related remodeling, including immunosenescence, chronic inflammation, stromal alteration, and metabolic shifts, the tumor microenvironment evolves in parallel, producing distinct patterns of tumor progression and therapy response (Erbe et al., 2021; Guegan et al., 2025). Yet, despite older adults comprising the majority of cancer diagnoses, they continue to be underrepresented in clinical research (Kao et al., 2025; Pallis et al., 2010; Berger et al., 2006). Emerging computational and image-based tools, as highlighted by the studies in this Research Topic, provide new opportunities to design age-inclusive strategies and overcome limitations in conventional risk stratification and biological understanding. These contributions collectively map the complex interplay between aging physiology and cancer behavior while showcasing the strengths and weaknesses of current analytical approaches.

## 2 Methodological integration: machine learning, radiomics, transcriptomics, and clinical modeling

### 2.1 Machine learning for risk prediction

Several studies in this Research Topic deploy machine learning (ML) to address gaps in traditional clinical prediction models. The differentiated thyroid cancer (DTC) study demonstrates the strength of ensemble algorithms, particularly XGBoost, for capturing nonlinear interactions between clinical variables. Similarly, the breast cancer axillary lymph node burden analysis highlights the value of combining radiomics with ML to extract predictive imaging signatures not discernible through human assessment. These methods show clear advantages in handling high-dimensional data, improving accuracy, and enabling personalized risk estimation. However, a shared limitation emerges: ML models rely heavily on retrospective datasets, are vulnerable to overfitting, and often lack interpretability despite tools like SHAP. External validation, present in some studies but absent in others, remains essential before these tools can be clinically deployed.

### 2.2 Transcriptomics and immunogenomic modeling

The study identifying T-cell exhaustion and macrophage polarization genes in breast cancer leverages multi-omics data integration and machine learning to uncover immune-related prognostic signatures. This contrasts with imaging-driven methods but shares the goal of revealing biological complexity underlying variable clinical outcomes.

While this approach offers mechanistic depth not available through radiomics or clinical modeling, it introduces its own constraints, such as variability across datasets, bulk RNA-seq limitations (cell-type averaging), and challenges in translating genetic signatures into actionable clinical decision-making.

### 2.3 Nomograms as clinically pragmatic tools

The glioma and colorectal neuroendocrine neoplasms (CRNEN) studies use traditional regression-based nomograms, long valued for their transparency and clinician usability. Unlike ML models, nomograms allow straightforward bedside application and highlight effect sizes for individual variables. Yet, these model types assume linearity and may oversimplify complex interactions, a trade-off between usability and nuance.

Notably, the CRNEN models incorporate a dual predictive-prognostic framework, illustrating how classic methodologies continue to evolve when embedded within large, high-quality datasets.

### 2.4 Clinical case evidence

The TAS-102 case report, while not methodologically complex, fills a crucial evidence gap: real-world therapeutic guidance for frail, multimorbid elderly patients. Such clinical narratives complement computational studies by grounding innovations in practical, human-centered contexts, an essential dimension often absent from data-driven work.

### 2.5 Cross-study themes: What do these findings tell us?

#### 2.5.1 Aging as a central yet underexplored modifier of cancer biology

Across tumor types, these works emphasize that aging fundamentally shapes cancer risk, microenvironment, treatment tolerance, and clinical trajectory. Yet, only a minority of the included studies explicitly stratify by age or examine age-specific biological mechanisms, revealing a critical research gap.

#### 2.5.2 The rise of precision oncology tools

Radiomics, machine learning, and transcriptomics each offer a different “lens” through which to view tumor behavior. When integrated, these modalities could enable more accurate, multi-dimensional prediction models that reflect both tumor biology and host factors, particularly in older patients.

#### 2.5.3 Data limitations and the need for prospective validation

Nearly all studies rely on retrospective datasets such as SEER or single-center cohorts. While rich in sample size, these datasets lack functional biomarkers, treatment toxicity data, and geriatric assessments. Prospective, age-inclusive cohorts are urgently needed.

### 2.6 Limitations across the Research Topic

Despite their contributions, the studies share several overarching limitations:

1. Retrospective designs introduce selection bias and limit causal inference.
2. Lack of functional aging measures: chronological age alone inadequately captures biological aging or treatment tolerance.
3. Limited longitudinal data: Few studies examine dynamic changes over time, especially relevant for aging-related biology.
4. Sparse diversity in many datasets underrepresents low-income, rural, and minority populations.
5. Translational gap: predictive models often lack pathways for real-world implementation in clinics.

Addressing these limitations will be essential to ensure that computational advances translate into meaningful clinical impact.

### 2.7 Future perspectives

#### 2.7.1 Integrating geriatric assessment with computational tools

Models incorporating frailty indices, functional status, inflammatory markers, and patient-reported outcomes could dramatically improve prediction accuracy for older adults.

#### 2.7.2 Multi-modal predictive platforms

Future studies should explore models that combine:

- Clinical data
- Radiomics
- Genomics
- Immune profiling
- Aging biomarkers

Such integration could capture the full biological and clinical complexity of cancer in aging populations.

#### 2.7.3 Prospective and interventional studies

Predictive models must move beyond validation toward prospective testing, where predictions actively inform treatment choices.

#### 2.7.4 Ethical and policy considerations

As ML models become more prevalent, transparency, fairness, and bias mitigation will be critical, particularly in elderly populations disproportionately affected by health disparities.

## 3 Conclusion

The studies in this Research Topic collectively demonstrate how emerging analytic tools and biological insights can refine cancer characterization and improve clinical decision-making across diverse tumor types. Yet they also highlight persistent challenges: inadequate age-specific data, limited external validation, and gaps in real-world applicability. Bridging these divides will require interdisciplinary collaboration, prospective validation, and a deeper commitment to studying cancer through the lens of aging biology.

By embracing these directions, the field can move toward genuinely personalized, age-inclusive oncology where innovations in cancer immunotherapy, computational science, and geroscience converge to benefit all patients across the lifespan.
